# A Novel Method for the Determination of Ganciclovir Triphosphate in Human Red Blood Cells by LC–MS/MS

**DOI:** 10.21627/6wt6r959

**Published:** 2026-05-29

**Authors:** Qiao-lan Xuan, Jun-tao Zheng, Jia-jia Yan, Long-shan Liu, Ji-feng Meng, Pan Chen, Jie Chen, Xiao Chen, Min Huang, Ke-jing Tang, Chang-xi Wang, Xia Zhu

**Affiliations:** Department of Pharmacy, the First Affiliated Hospital, Sun Yat-sen University, Guangzhou, China; Institute of Clinical Pharmacology, School of Pharmaceutical Sciences, Sun Yat-sen University, Guangzhou, China; Institute of Precision Medicine, the First Affiliated Hospital, Sun Yat-sen University, Guangzhou, Guangdong, China; Department of Pharmacy, the First Affiliated Hospital, Sun Yat-sen University, Guangzhou, China; Organ Transplant Center, the First Affiliated Hospital, Sun Yat-sen University, Guangzhou, China; Department of Pharmacy, the First Affiliated Hospital, Sun Yat-sen University, Guangzhou, China; Institute of Clinical Pharmacology, School of Pharmaceutical Sciences, Sun Yat-sen University, Guangzhou, China; Department of Pharmacy, the First Affiliated Hospital, Sun Yat-sen University, Guangzhou, China; Department of Pharmacy, the First Affiliated Hospital, Sun Yat-sen University, Guangzhou, China; Department of Pharmacy, the First Affiliated Hospital, Sun Yat-sen University, Guangzhou, China; Institute of Precision Medicine, the First Affiliated Hospital, Sun Yat-sen University, Guangzhou, Guangdong, China; Department of Pharmacy, the First Affiliated Hospital, Sun Yat-sen University, Guangzhou, China; Organ Transplant Center, the First Affiliated Hospital, Sun Yat-sen University, Guangzhou, China; Department of Pharmacy, the First Affiliated Hospital, Sun Yat-sen University, Guangzhou, China

**Keywords:** ganciclovir, ganciclovir-triphosphate, LC-MS/MS, renal transplant recipients

## Abstract

**INTRODUCTION:**

Ganciclovir (GCV) and its prodrug valganciclovir (VGCV) are commonly used to prevent and treat cytomegalovirus (CMV) infections in solid organ transplant recipients. Because of high interindividual pharmacokinetic variability, therapeutic drug monitoring (TDM) is important for optimizing therapy. Ganciclovir-triphosphate (GCV-TP), the active intracellular metabolite, is linked to both efficacy and toxicity; however, clinical monitoring is limited by analytical challenges.

**OBJECTIVES:**

To develop and validate a simple and sensitive liquid chromatography–tandem mass spectrometry (LC–MS/MS) method for quantifying GCV-TP in human red blood cells (RBCs).

**METHODS:**

GCV-TP was extracted using protein precipitation, separated on a BioBasic AX column, and detected with tandem mass spectrometry using guanosine-^13^C_10_-5′-triphosphate as the internal standard.

**RESULTS:**

The assay was linear over 0.01–2.00 μg/mL (*r*^2^ ≥ 0.99), with intra- and inter-assay variability ≤7.99%. GCV-TP in RBC lysate was stable when stored at room temperature for 4 h or at 4 °C for 24 h. This method was applied to RBC samples from 27 renal transplant recipients receiving VGCV, with a median GCV-TP concentration of 180.8 pmol/8 ×10^9^ RBC (IQR: 113.6–297.6 pmol/8 ×10^9^ RBC). GCV-TP levels showed a significant correlation with VGCV dose (*p* = 0.020, *r* = 0.31) but not with plasma GCV concentrations (*p* > 0.05).

**CONCLUSION:**

This validated method enables reliable measurement of intracellular GCV-TP and may serve as a useful tool to support individualized VGCV therapy and pharmacodynamic monitoring in clinical practice.

## Non-Specialist Summary

Ganciclovir and valganciclovir prevent and treat CMV in transplant patients; however, standard blood tests may not accurately reflect intracellular drug efficacy. This study developed a simple, reliable method to measure ganciclovir-triphosphate—the active form of the drug—inside red blood cells. When the method was applied to kidney transplant patient samples, the results showed that intracellular levels correlate with the prescribed valganciclovir dose but not with plasma ganciclovir concentrations. This method may help clinicians better individualize therapy and improve monitoring.

## 1. Introduction

Cytomegalovirus (CMV), the most common viral infection after solid organ transplant (SOT), causes substantial morbidity and mortality [1, 2]. Among SOT recipients, CMV infection has been associated with reduced long-term patient survival, increased susceptibility to opportunistic infections, allograft dysfunction, acute and chronic graft rejection, and increased total costs [3, 4]. Ganciclovir (GCV) and its oral prodrug, valganciclovir (VGCV), both of which are acyclic guanine nucleoside analogs, are the first-line drugs of prophylaxis and treatment for CMV infection in the post-transplant setting [5].

Neither GCV nor VGCV exhibits intrinsic activity; both require intracellular activation through a multistep enzymatic process to exert an antiviral effect or cytotoxicity (Figure 1) [6]. VGCV is first hydrolyzed to GCV by intestinal and hepatic esterases. In CMV-infected cells, GCV is then converted to its monophosphate form (GCV-MP) by the viral kinase pUL97; this step is rate-limiting because of its reliance on viral enzyme activity. Subsequent phosphorylation by cellular kinases yields the active triphosphate form (GCV-TP) [7–9]. Structurally resembling deoxyguanosine triphosphate (dGTP), GCV-TP is preferentially incorporated by viral DNA polymerase, leading to chain termination, disruption of viral DNA synthesis, and inhibition of viral replication [10]. NUDT15 (nucleoside diphosphate–linked moiety X-type motif 15) was recently proved to inactivate GCV-TP by the diphosphatase, which may limit the antiviral effect against CMV in vitro [11].

**Figure 1.**
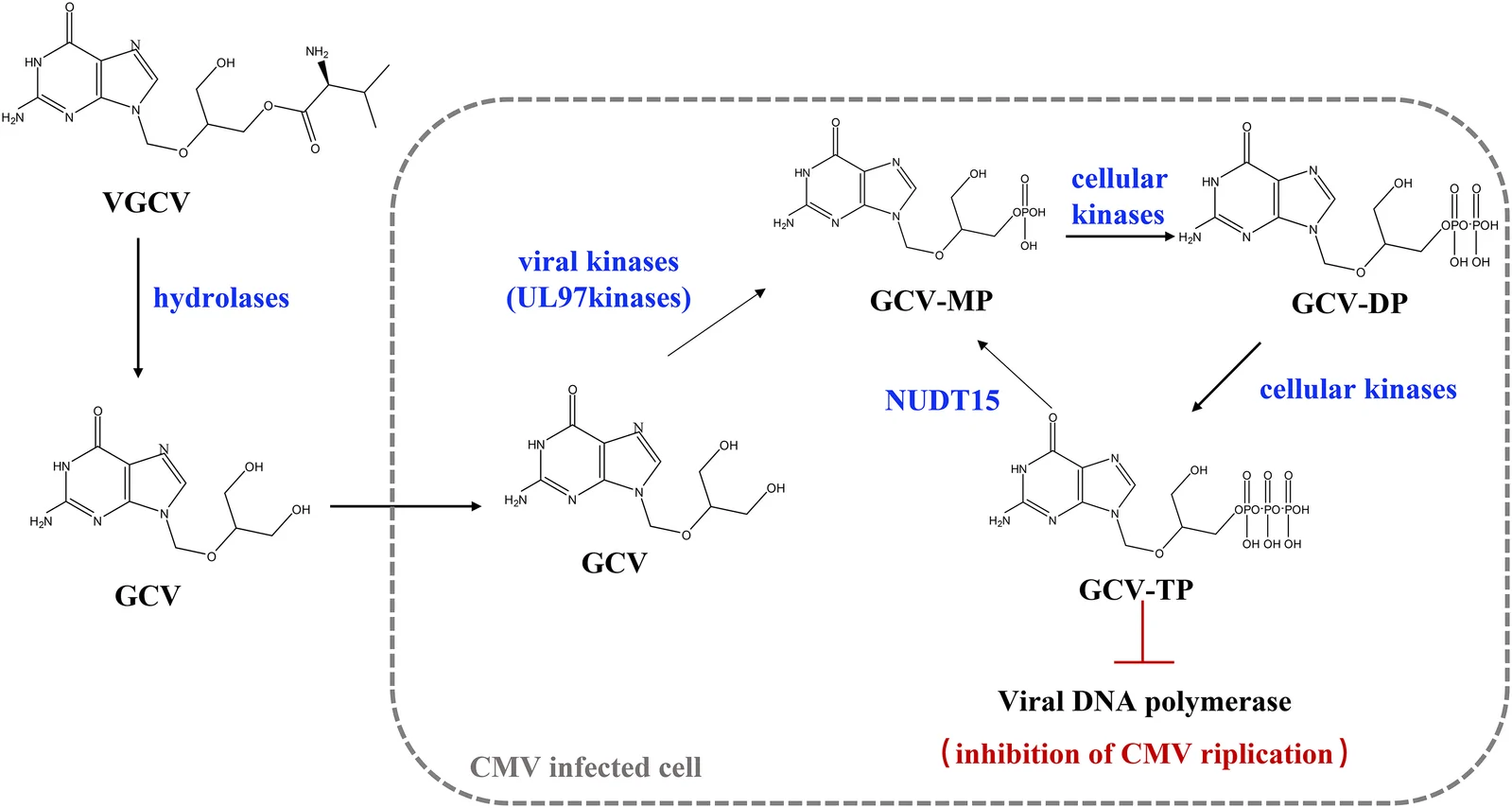
Metabolism of ganciclovir (GCV) and valganciclovir (VGCV). GCV-MP, ganciclovir-monophosphate; GCV-DP, ganciclovir-diphosphate; GCV-TP, ganciclovir-triphosphate; NUDT15, nucleoside diphosphate-linked moiety X-type motif 15; CMV, cytomegalovirus.

GCV/VGCV are highly effective anti-CMV agents but are associated with severe dose-dependent adverse effects, particularly myelotoxicity [12, 13]. As many as 50% of individuals administered GCV/VGCV experienced hematological toxicity (particularly neutropenia), which may lead to premature discontinuation of GCV/VGCV or to lowering of the dose [14–16]. Many laboratories around the world detect ganciclovir exposure in plasma to monitor therapeutical effect and toxicity [17]. However, no conclusive correlation between GCV levels and neutropenia was found in numerous clinical studies [12, 18–22]. This lack of correlation may be, in part, caused by GCV not reflecting individual levels of GCV-TP in vivo [20, 23]. This assumption is corroborated by a recent study demonstrating that GCV-TP in peripheral blood mononuclear cell (PBMC), but not GCV in plasma, was associated with the decreased neutrophil count [20]. However, PBMC isolation is complex and costly. Achieving sufficient sensitivity for drug quantification often requires a relatively large volume (5 mL) of whole blood, increasing the burden on patients [23, 24]. Red blood cells (RBCs) are present in abundance and easy to separate, making them the logical choice as a surrogate matrix [25–27]. For the purine analogues as thiopurines, detecting their tri-phosphorylate in RBCs has been reported to be a helpful strategy to analyze their clinical effect during therapy [28].

Thus, in the present study, we first develop a fast and sensitive LC–MS/MS method for the direct measurement of GCV-TP in human RBCs and then apply this method in patients given VGCV as part of their treatment.

## 2. Methods

### 2.1. Chemicals and Reagents

GCV-TP was acquired from Jena Bioscience (NU-275, Jena, Germany); guanosine-^13^C_10_-5′-triphosphate (internal standard, IS) was acquired from Santa Cruz Biotechnology (SC-300776, Dallas, Texas, USA). Ethylenediaminetetraacetic acid (EDTA) solution (disodium salt, dihydrate) was prepared as a 50 mmol/L solution and adjusted to pH 10.5 with 5 N NaOH solution. Pure water was obtained from a Milli-Q system (Millipore, Germany).

### 2.2. Preparation of Standards, Internal Standards, and Quality Controls

Stock solutions of GCV-TP and IS were prepared in pure water at a final concentration of 500 μg/mL and stored at –80 °C. Working solutions of GCV-TP at final concentrations of 0.05, 0.10, 0.25, 0.50, 1.00, 2.50, 5.00, and 10.00 μg/mL and 1.00 μg/mL for IS were prepared by dilution with EDTA solution. Internal quality control (IQC) samples were prepared at 0.10, 0.80, and 8.00 μg/mL with EDTA solution.

### 2.3. Blood Sampling

Patients had been on stable GCV/VCGV dosage for at least 1 month prior to the study. The study protocol was approved by the ethics committee of the First Affiliated Hospital of Sun Yat-sen University (No. 2024ZSLYEC-713). All patients provided written informed consent.

### 2.4. Preparation of Erythrocyte Lysate and Sample Work-Up Procedure

Blood samples were collected in EDTA tubes and immediately stored at 4 °C. Packed RBCs and plasma were separated within 8 h after blood collection by centrifugation at 4000 rpm for 10 min at 4 °C. The packed RBCs were subsequently washed three times with an equal volume of chilled PBS. A 100 μL aliquot of packed RBCs was suspended in 200 μL of PBS for determination of the RBC count (BC-6800 Plus, Mindray, China). The remaining RBCs were mixed with EDTA solution (50 μL RBCs with 240 μL EDTA) to obtain RBC lysates. The RBC lysates were stored at –80 °C until further analysis.

Prior to analysis, patient RBC lysates (290 μL) were thawed at room temperature, spiked with 10 μL of IS (1 μmol/L), and vortex-mixed. Calibration standards and quality control (QC) samples were prepared by adding 10 μL of the appropriate working solution and 10 μL of IS (1 μmol/L) to blank RBC lysate (50 μL RBCs with 230 μL EDTA), followed by vortex mixing. The calibration curve was constructed at nominal concentrations of 0.01, 0.02, 0.05, 0.10, 0.20, 0.50, 1.00, and 2.00 μg/mL. QC samples were prepared at low, medium, and high concentrations of 0.02, 0.16, and 1.60 μg/mL, respectively.

For sample pretreatment, 50 μL of methanol and 250 μL of dichloromethane were added as protein precipitants, followed by thorough mixing for 10 min. The mixture was then centrifuged at 15,000 rpm for 20 min at 4 °C. A 140 μL aliquot of the supernatant was transferred to a clean microcentrifuge tube, and 60 μL of acetonitrile was added to ensure the same proportion as the liquid phase. Five microliters of supernatant was injected for analysis. The post-treatment samples were placed in an auto-sampler at 10 °C before testing. For clinical application and data comparison with other clinical research, GCV-TP concentrations (μg/mL) were converted to pmol/8 ×10^8^ RBC using the formula


$$\text{GCV}-\text{TP}\left(\text{pmol}/8\times 10^{9}\text{RBC}\right)=\frac{\text{GCV}-\text{TP}\left(\text{µg}/\text{mL}\right)\times 4\times 10^{6}}{\text{molecular weight of GCV}-\text{TP }\left(495.17\text{µg}/\text{µmol}\right)\times \text{RBC counts}\times 1.5}$$

### 2.5. LC–MS/MS Conditions

An AB SCIEX 7500 triple-quadrupole mass spectrometer equipped with an electrospray ion source (AB SCIEX, MA, USA) was coupled to a Shimadzu Nexera LC-40 HPLC system. The ionization mode of the mass spectrometer was positive electrospray ionization (ESI). Nitrogen was used as an ion source gas 1, gas 2, curtain gas, and collision gas. The capillary voltage was set at 3500 V, and the source temperature was set at 200 °C. The ion source gas 1, gas 2, and curtain gas pressures were sequentially set as 35 psi (241 kPa), 70 psi (483 kPa), and 40 psi (276 kPa), respectively. The mass spectrometer was operated in multiple reaction monitoring (MRM) mode. Fragmentor voltages and collision energies for each transition were optimized by a series of flow injections of pure standards. MRM transitions under the optimized conditions are summarized in Table 1.

**Table 1.** Mass parameters of the analytes in multiple reaction mode

Analyte	Precursorion (m/z)	Quantifier		Qualifier		Retention time (min)
		Production (m/z)	Collision energy (eV)	Production (m/z)	Collision energy (eV)	
GCV-TP	496.05	151.98	31	166.95	37	5.40
Guanosine-^13^C_10_-5′-TP	534.04	157.00	31	139.98	95	5.44

Chromatographic separation was carried out at 35 °C on a BioBasic AX column (2.1 × 50 mm^2^, 5 μm particle size; Thermo Electron, Egelsbach, Germany). The mobile phases were composed of water:acetonitrile (7:3, v/v) containing 10 mM ammonium acetate at pH 5.5 for phase A, and 1 mM ammonium acetate at pH 10.5 for phase B. The gradient elution was initiated at 10% phase B. From 1.5 to 2.0 min, phase B was increased to 50% and maintained at this level until 3.5 min. It was then increased to 90% phase B between 3.5 and 4.0 min and maintained at 90% until 6.5 min. From 6.5 to 6.51 min, phase B was decreased back to 10% and held constant until 8.0 min. The flow rate was 0.25 mL/min.

### 2.6. Method Validation

The method [including selectivity, the lower limit of quantification (LLOQ), carry-over effect, linearity, accuracy, precision, recovery, matrix effect, and stability] was validated according to published guidelines [29].

#### 2.6.1. Selectivity and LLOQ

Selectivity was evaluated by analyzing RBC samples obtained from six individual donors. The peak areas observed in blank samples were compared with those at the LLOQ. Interference was considered acceptable if the response in blank samples was less than 20% of the LLOQ response for GCV-TP and less than 5% for the IS. Sensitivity was determined as the lowest concentration with a signal-to-noise (S/N) ratio greater than 10.

#### 2.6.2. Carry-over effect and linearity

To assess carry-over, a blank RBC sample was injected immediately after the highest calibration standard. The calibration curve for GCV-TP (0.01–2.00 μg/mL) was generated by plotting the peak-area ratio between GCV-TP and the IS versus the nominal concentration, using a 1/*X* weighting factor. The curve was accepted if the accuracy of each standard was within ±15% (±20% for LLOQ) and the correlation coefficient (*r* ^2^) exceeded 0.980.

#### 2.6.3. Intra-day and inter-day precision and accuracy

Quality control (QC) samples were prepared in blank RBCs at GCV-TP concentrations of 0.02, 0.16, and 1.60 μg/mL. Intra- and inter-day accuracy and precision were assessed by analyzing six replicates at each level over three separate days. Precision and accuracy were considered acceptable if within ±15% for QC samples and within ±20% for LLOQ samples.

#### 2.6.4. Recovery and matrix effect

Recovery was determined by comparing the peak areas of analytes spiked into RBCs before and after extraction. The matrix factor (MF) was calculated as the ratio of the peak area for extracted RBC samples to that for aqueous standards at equivalent concentrations. The IS-normalized MF was obtained by dividing the MF of GCV-TP by that of the IS. The coefficient of variation (CV) of the IS-normalized MF was required to be <15%. All assessments were performed in six replicates at each QC and LLOQ level.

#### 2.6.5. Stability

The stability of GCV-TP in whole blood was evaluated in ETDA blood from six patients; the samples were stored at 4 °C for as long as 24 h. The stability of GCV-TP during sample processing was assessed by spiking the analyte into blank RBC lysates, with six replicates at each QC level. Short-term stability was tested in RBCs at room temperature and at 4 °C for 24 h. Freeze–thaw stability was evaluated after three cycles at –80 °C. Long-term stability was assessed in RBC lysates stored at –80 °C for 60 days. Processed sample stability was examined by re-injecting extracts stored in the autosampler at 10 °C for 24 h.

### 2.7. Clinical Application

Twenty-seven patients treated with VGCV for at least 4 weeks were included in this study. One milliliter of venous blood sample was collected for extraction of RBCs and plasma. Both the RBC lysate and plasma were stored at –80 °C until analysis. Plasma was used to measure the GCV trough concentration (*C*_trough_), whereas the RBC lysate was used to determine the GCV-TP concentration.

### 2.8. Plasma GCV Measurements

Briefly, plasma samples (100 μL) were prepared by protein precipitation with acetonitrile (300 μL), followed by analysis using a 1260 HPLC system with a 6420 triple-quadrupole mass spectrometer (Agilent, USA). Chromatographic separation was achieved on a Hypersil Gold C18 column with a mobile phase consisting of 0.1% formic acid in water (A) and methanol (B) at a flow rate of 0.4 mL/min. Ganciclovir was detected in positive-ion mode using multiple reaction monitoring (MRM), with the transition of m/z 256.01–152.00. The calibration curve for plasma ganciclovir was linear over the concentration range 0.04–20.00 μg/mL, with *r* ^2^ ≥ 0.9991. Intra- and inter-day accuracy and precision were within acceptable limits (bias within ±15% and coefficient of variation ≤15%). Plasma and RBC samples were collected at the same visit to ensure comparability between plasma ganciclovir and intracellular GCV-TP measurements.

### 2.9. Statistical Analysis

Statistical analyses and calculations were performed with the SPSS 21.0 software (SPSS, Chicago, IL, USA) and GraphPad Prism 9 software (GraphPad, La Jolla, CA, USA). Statistical descriptions of the quantitative variables were expressed as the median and interquartile range (IQR). Spearman’s correlation coefficient was used to evaluate correlations between GCV-TP levels and VGCV dose or GCV levels in plasma. A *p*-value less than 0.05 was considered to indicate statistically significant results.

## 3. Results

### 3.1. Selectivity and LLOQ

No significant matrix interferences were observed in the m/z transitions of GCV-TP and the IS across RBC samples from six donors. Figure 2 shows a representative blank chromatogram from one donor. In addition, no crosstalk was detected between GCV-TP and the IS. The S/N ratio at the LLOQ was 89.0 for GCV-TP.

**Figure 2.**
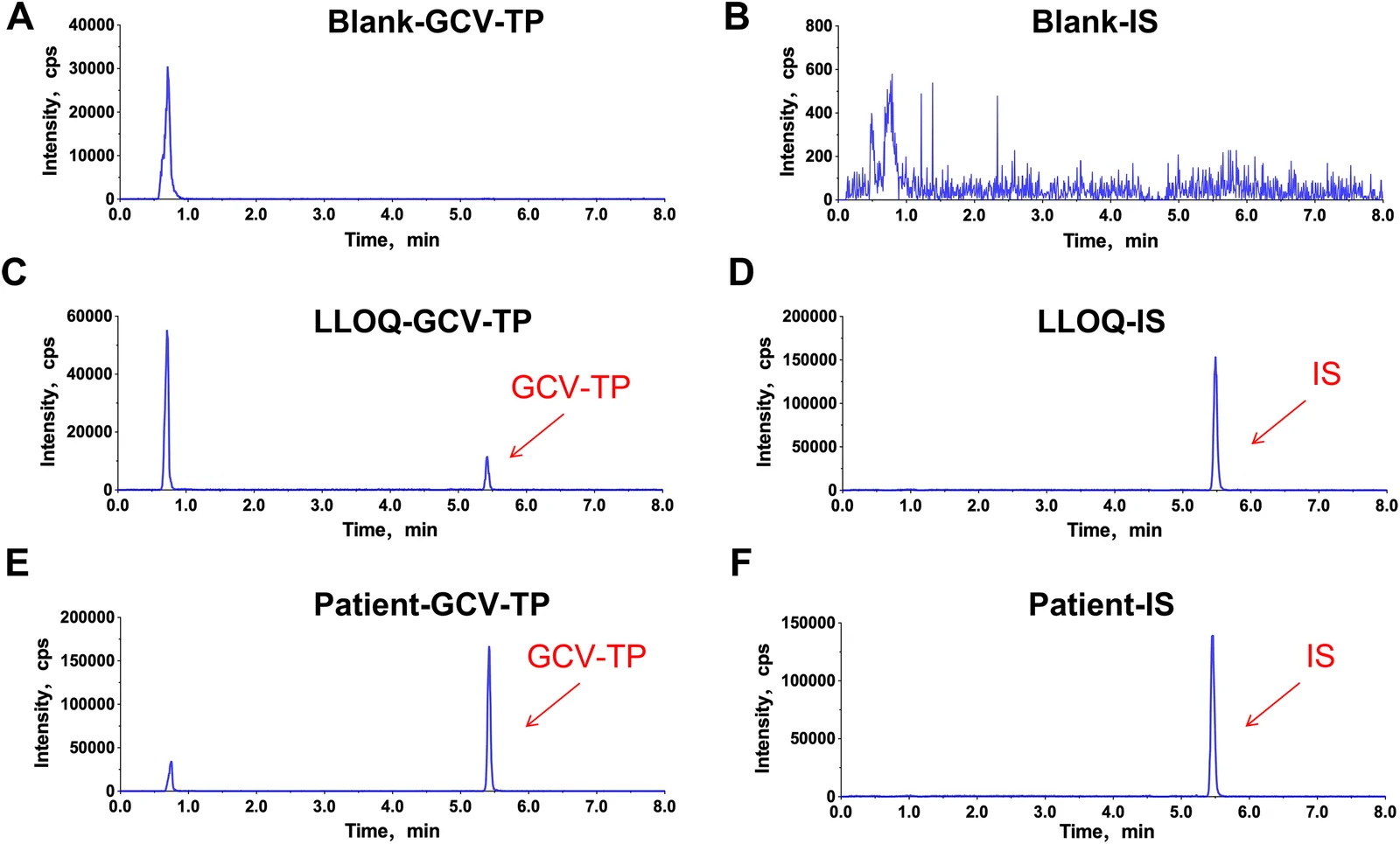
Representative chromatograms of blank RBC samples (A, B); blank RBC samples spiked with GCV-TP at the LLOQ and the IS (C, D), and a representative patient RBC sample containing GCV-TP and the IS (E, F).

### 3.2. Carry-Over Effect and Linearity

No significant carry-over was observed when blank RBC samples were injected immediately after the highest calibration standard (with IS), with signal intensities ≤20% of the LLOQ and ≤5% of the IS. Calibration curves were constructed on three separate days, each comprising eight concentration levels. The curves were generated by plotting the peak-area ratio (*y*) versus the analyte concentration (*x*) using linear regression with 1/*X* weighting. The GCV-TP calibration curves showed good linearity, with *r*^2^ > 0.99.

### 3.3. Intra-Day and Inter-Day Precision and Accuracy

Intra- and inter-day precision and accuracy were evaluated using six replicates at each QC level and the LLOQ, as summarized in Table 2. Intra-day precision ranged from 3.33% to 7.99%, with accuracy between 94.75% and 101.69%. Inter-day precision ranged from 3.88% to 6.69%, and accuracy from 97.00% to 101.23%.

**Table 2.** Accuracy and precision of ganciclovir triphosphate in red blood cells.

QC levels (μg/mL)	Intra-day (*n* = 6)	Accuracy (%)	Inter-day (*n* = 18)	Accuracy (%)
	CV (%)		CV (%)	
0.01	7.99	97.24	6.69	97.00
0.02	3.33	94.75	5.14	97.10
0.16	6.39	101.69	3.88	101.07
1.60	4.15	99.64	5.62	101.23

### 3.4. Recovery and Matrix Effect

A comparison between neat standards and RBC-extracted standards of GCV-TP was conducted to assess recovery and matrix effects. The RBC recoveries of GCV-TP at LQC, MQC, and HQC levels were 87.50%, 91.59%, and 96.27%, respectively, with coefficients of variation (CVs) all less than 4.25% (Table 3). The IS-normalized matrix factors (MFs) of GCV-TP at low, medium, and high concentrations were 76.70%, 74.87%, and 66.14%, respectively (Table 3). The CVs of the IS-normalized MFs were all less than 8.95%, indicating acceptable matrix effects with consistent ion suppression.

**Table 3.** Matrix effect and extraction recovery of ganciclovir triphosphate in red blood cells.

QC levels (μg/mL)	Extraction recovery (%)	CV (%)	Matrix effect (%)	CV (%)
0.01	80.33	8.98	73.59	11.48
0.02	87.50	4.25	76.71	4.11
0.16	91.59	4.72	74.87	8.95
1.60	96.27	5.22	66.14	4.93

### 3.5. Stability

The results showed GCV-TP concentrations remained stable when blood samples were stored at 4 °C immediately after collection from six independent patients (Figure 3). Thus, preparation of packed RBC within 8 h after blood sampling appears to be reliable. In this study, the stability of GCV-TP in RBC lysate (EDTA, pH = 10.5) was assessed under short- and long-term storage conditions, including 4 h at room temperature, 24 h at 4 °C, and 60 days at –80 °C (Table 4). GCV-TP was not affected across all conditions, indicating good analyte stability during sample handling and storage. Meanwhile, three freeze–thaw cycles did not affect the GCV-TP concentrations. Processed samples were stable in an autosampler for 24 h at 10 °C (Table 4).

**Figure 3.**
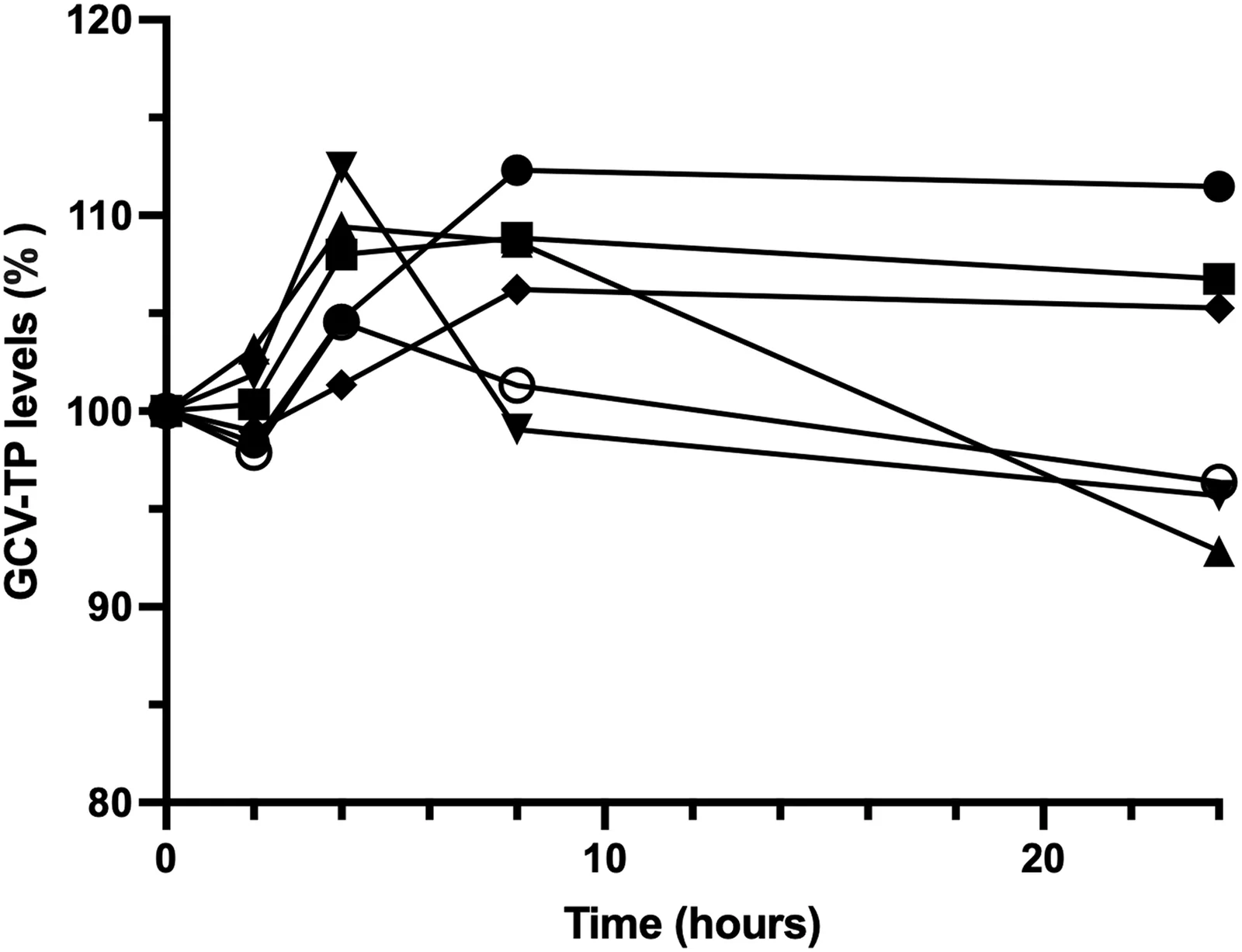
Stability of ganciclovir-triphosphate in blood. Individual ganciclovir-triphosphate levels in red blood cells (RBCs) from blood samples stored at 4 °C before isolation of RBCs for 24 h are shown for six patients treated with valganciclovir.

**Table 4.** Stability of ganciclovir triphosphate in red blood cells under different conditions.

QC levels (μg/mL)	Short-term stability at 4 ℃ for 24 h (*n* = 6)		Short-term stability at 25 ℃ for 4 h (*n* = 6)		Extraction stability in autosampler for 24 h (*n* = 6)		Long-term stability at –80 ℃ for 60 days (*n* = 6)		Three freeze-thaw stability at –80 ℃ (*n* = 6)	
	Accuracy (%)	CV (%)	Accuracy (%)	CV (%)	Accuracy (%)	CV (%)	Accuracy (%)	CV (%)	Accuracy (%)	CV (%)
0.01	95.54	2.40	93.66	4.53	85.61	6.21	109.61	2.13	91.42	3.99
0.02	110.53	2.49	90.00	3.98	103.63	3.74	99.01	4.59	109.53	1.75
0.16	104.83	2.13	86.25	2.62	100.64	3.38	101.25	1.99	104.24	3.28
1.60	106.86	2.16	87.63	3.99	101.62	3.05	100.04	1.99	107.46	2.64

### 3.6. Clinical Application

In this study, 56 GCV-TP concentrations were obtained from 27 adult renal transplant patients who had been receiving a stable dose of VGCV for at least 4 weeks. Demographic and clinical data for the patients are summarized in Table 5. The GCV-TP was present at quantifiable levels by LC–MS/MS in all of the samples, with a median level of 185.2 pmol/8 ×10^9^ RBC (IQR: 119.2–280.4 pmol/8 ×10^9^ RBC) [0.10 µg/mL (IQR: 0.073–0.16 µg/mL)]. A moderate positive correlation was observed between VGCV dose and intracellular GCV-TP concentrations (*r* = 0.31, *p* = 0.020) (Figure 4B). No correlation was found between GCV *C*_trough_ and GCV-TP concentrations (*p* = 0.66) (Figure 4C).

**Figure 4.**
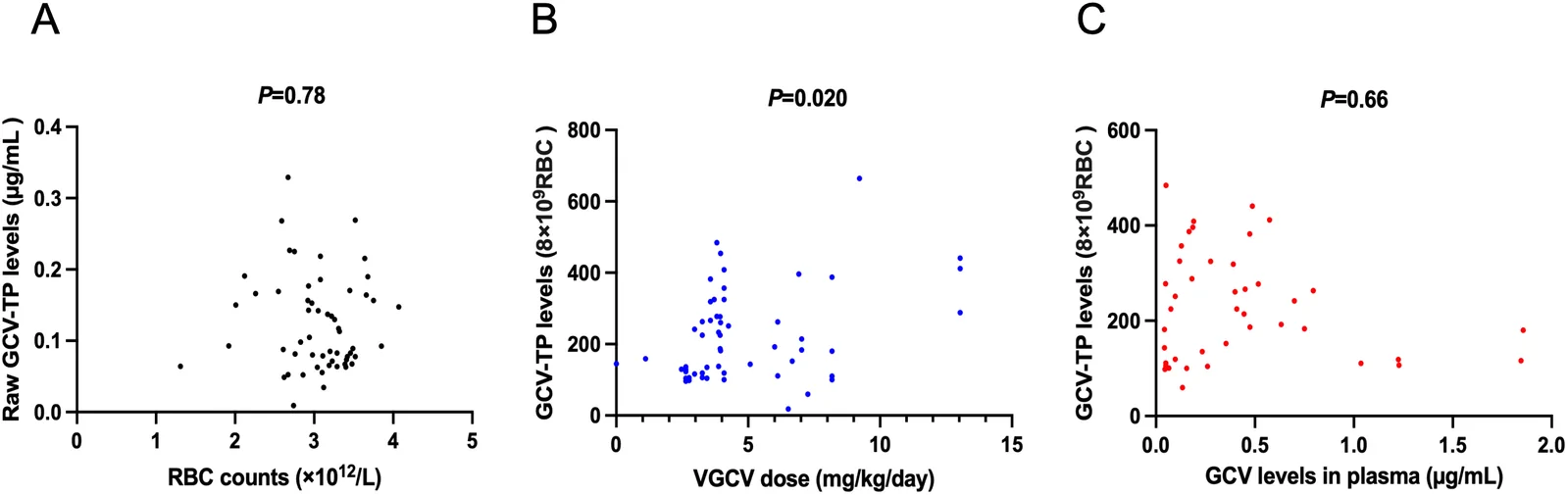
Relationship between raw ganciclovir triphosphate levels and RBC counts (A); ganciclovir triphosphate levels and valganciclovir dose (B); ganciclovir triphosphate levels and ganciclovir levels in plasma (C).

**Table 5.** Baseline characteristics of 27 Chinese renal transplant recipients

Number	Sex	Age (years)	Weight (kg)	Drug	Dose (mg)	Frequency	Body weight–normalized dose (mg/(kg·d))
1	Female	53	52	VGCV	450	TIW	3.71
2	Male	32	55	VGCV	450	QOD	4.09
3	Male	29	58	VGCV	225	TIW	1.11
4	Male	67	64	VGCV	450	QD	7.03
5	Male	24	55	VGCV	450	QD	8.18
6	Female	26	38	VGCV	450	TIW	5.08
7	Female	33	57	VGCV	450	QOD	3.95
8	Male	42	70	VGCV	450	TIW	2.76
9	Male	51	48.8	VGCV	450	QD	9.22
10	Male	60	69	VGCV	450	QD	6.52
11	Male	66	75	VGCV	450	QD	6.00
12	Female	42	34.5	VGCV	450	QD	13.04
13	Male	41	73	VGCV	450	TIW	2.64
14	Male	32	55	VGCV	450	QD	8.18
15	Male	27	59	VGCV	450	QOD	3.81
16	Female	34	65	VGCV	450	TIW	2.97
17	Male	37	69	VGCV	450	QOD	3.26
18	Male	28	67.5	VGCV	450	QD	6.67
19	Male	27	63	VGCV	225	QD	3.57
20	Male	44	78	VGCV	450	TIW	2.47
21	Male	28	56	VGCV	450	TIW	3.44
22	Male	51	65	VGCV	450	QD	6.92
23	Female	32	58	VGCV	450	QOD	3.88
24	Male	37	53	VGCV	225	QD	4.25
25	Male	39	73.5	VGCV	450	QD	6.12
26	Male	34	62	VGCV	450	QD	7.26
27	Female	29	49	VGCV	450	TIW	3.94

TIW: three times a week; QOD: every other day; QD: once daily

## 4. Discussion

In this study, we developed and validated a novel, simple, sensitive, and reproducible LC–MS/MS method for quantifying GCV-TP in human RBCs and demonstrated its applicability in clinical samples from patients receiving VGCV. Importantly, this method overcomes a key limitation of conventional TDM by enabling the assessment of intracellular exposure to the active antiviral metabolite.

GCV-TP, the active metabolite of GCV and VGCV, is implicated in myelosuppression through the inhibition of DNA polymerase in hematopoietic progenitor cells [10]. Its intracellular exposure has been associated with neutropenia in renal transplant recipients [19]. In contrast, plasma GCV concentrations do not reliably reflect intracellular GCV-TP levels [20], consistent with our findings, highlighting the limitations of plasma-based TDM. This discrepancy may be partly explained by interindividual differences in intracellular metabolism, particularly NUDT15 activity, because reduced-function variants can promote intracellular GCV-TP accumulation and increase the risk of myelosuppression during VGCV prophylaxis [11, 30]. Although PBMCs are the primary target cells, their clinical use is limited by laborious isolation procedures and the requirement for larger blood volumes [23]. In contrast, RBCs are more accessible and practical for routine analysis. Therefore, the RBC-based high-throughput method established in this study may provide a feasible approach for monitoring intracellular GCV-TP exposure and better evaluating the risk of hematologic toxicity.

Antiviral metabolites can be quantified using either direct or indirect measurement methods [23]. The indirect approach involves solid-phase extraction of metabolite fractions, followed by enzymatic or chemical conversion into the parent compound prior to analysis, which is both time-consuming and costly. To date, only one method has been reported for detecting GCV-TP in human PBMCs, as described by Billat et al. [24]. In contrast to their indirect approach, we adopted a novel direct quantification strategy to simplify sample preparation and enhance efficiency.

A major challenge in the direct quantification method was preventing the in vitro conversion between di- and triphosphate forms. A previous study demonstrated that adding 50 mM EDTA at a high pH (10.5) inhibits this conversion by chelating magnesium ions in 6-thioguanosine triphosphate [31]. In the present study, the stability of GCV-TP in RBC lysates (with EDTA at pH 10.5) was assessed under various conditions, demonstrating excellent analyte stability during sample handling and storage. Chromatographic separation was another key innovation, achieved using a weak anion-exchange column with a pH gradient ranging from 5.5 to 10.5 and a decreasing ammonium acetate concentration (from 10 mM to 1 mM). Compared with other conditions, the multi-step gradient not only reduced retention time but also improved peak symmetry [23, 25].

Despite the successful validation and clinical application of this assay, certain limitations must be acknowledged. First, because no commercial isotopically labeled GCV-TP is currently available, guanosine-^13^C_10_-5′-triphosphate was used as a structural analog. Although it is not an exact isotopic match for the analyte, this compound shares a highly similar triphosphate moiety and polar characteristics, ensuring comparable behavior during weak anion-exchange chromatography and ESI. The suitability of this IS was confirmed by the consistency of the IS-normalized matrix factors, all of which exhibited CVs less than 10.59%. Second, direct evidence linking specific RBC GCV-TP concentration thresholds to clinical outcomes—such as CMV viral load reduction or the severity of neutropenia—remains limited. While RBCs serve as a stable and accessible surrogate matrix, they lack nuclei and may not perfectly reflect the pharmacodynamics occurring in nucleated target cells, where viral DNA polymerase inhibition primarily occurs. Therefore, this study provides a necessary methodological foundation. Future prospective trials are warranted to compare GCV-TP levels in RBCs and PBMCs within the same patient cohorts to define clear therapeutic windows and clarify the translational relevance of RBC-based measurements for optimizing clinical outcomes.

## 5. Conclusion

This validated method provides a reliable and quantifiable means of measuring GCV-TP in RBCs and represents a potentially useful tool for future clinical studies evaluating the relationship between intracellular GCV-TP exposure, therapeutic efficacy, toxicity, and pharmacogenetic variability in patients receiving GCV/VGCV therapy.

## Data Availability

The datasets generated and analyzed during the current study are available from the corresponding authors upon reasonable request.

## Abbreviations

CMV: Cytomegalovirus
CV: Coefficient of Variation
dGTP: Deoxyguanosine Triphosphate
GCV: Ganciclovir
GCV-MP: Ganciclovir-Monophosphate
GCV-TP: Ganciclovir-Triphosphate
HQC: High Quality Control
IS: Internal Standard
LC–MS/MS: Liquid Chromatography–Tandem Mass Spectrometry
LLOQ: Lower Limit of Quantitation
LQC: Low Quality Control
MF: Matrix Factor
MRM: Multiple Reaction Monitoring
MQC: Medium Quality Control
NUDT15: Nucleoside Diphosphate–Linked Moiety X-Type Motif 15
PBMC: Peripheral Blood Mononuclear Cell
RBC: Red Blood Cell
SOT: Solid Organ Transplant
TDM: Therapeutic Drug Monitoring
TGTP: 6-Thioguanosine-5′-Triphosphate
VGCV: Valganciclovir
